# Microsoft teams virtual handover system

**DOI:** 10.1192/bjo.2021.559

**Published:** 2021-06-18

**Authors:** Mohit Mohan, Ruth Scally, James Reed, Calin Cavaropol

**Affiliations:** Birmingham and Solihull Mental Health NHS Foundation Trust

## Abstract

**Aims:**

Accurate and timely handover of clinical information is of great importance to continuity and safety of care. Psychiatry doctors typically cover a number of sites across a catchment when they are on-call. Consequently, handover between on-call teams and day teams in psychiatric hospitals is reliant on using the nursing staff as an intermediary to flag concerns or relying on the day teams proactively checking the notes on daily basis for outstanding tasks.

The key objective of this project was to use Microsoft teams to establish a handover system that is efficient, safe, reliable, easy to use and replicable.

**Method:**

A microsoft teams group was created comprising of all the medical staff members working at inpatient units across three sites that are part of Birmingham and Solihull Mental Health Trust. These members were divided into two groups - the ‘on-call team’ and the ‘day team’. Within the ‘day team’, every consultant was grouped with their junior doctors to form multiple subgroups.

A system was established wherein the two teams could communicate with each other by posting a message and tagging the appropriate team. A provision was made to create a channel for every ward to allow for easy segregation and monitoring of tasks.

Qualitative information about the use of the tool was monitored by monthly focus group meetings. A formal review of the messages was conducted after 8 weeks to assess the following parameters:

Number of messages posted

Number of messages acknowledged

Number of safety-related incidents

**Result:**

Initial evaluation of the results suggests that the new handover system was perceived to be safe, accurate and efficient while being intuitive and hassle-free. This increased the quantity and enhanced the quality of communication between the ‘on-call’ and the ‘day teams’ and allowed for early completion of tasks while reducing the number of safety-related incidents.

**Conclusion:**

The Microsoft teams proved to be a viable alternate tool to create a virtual handover process that is efficient, safe, reliable and user-friendly. It also has the potential to enhance the communication between inpatient and community teams.

